# Supplementary material for: Burnout among labor and birth providers in northern Tanzania: A mixed-method study
Source: medRxiv. 2023 Jun 3:2023.05.28.23290395. Preprint. [Version 1] doi: 10.1101/2023.05.28.23290395 (PMC10312826; doi:10.1101/2023.05.28.23290395)
Supplement: Supplementary file 1 — S1. Consort diagram [file media-1.zip › S1 Figure 1. Consort diagram.docx]

Supplemental table 1. Consort diagram

**Invited to participate**

**(n=60)**

Changed jobs (n=1)

Maternity leave (n=1)

Busy with clinical duties (n=2)

Loss to follow up (n=1)

**Attended *In Situ* training**

**(n=55)**

**Completed Immediate Post Assessment**

**(n=60)**

**Attended MAMA training**

**(n=60)**

**Completed 3 Month Assessment**

**(n=59)**

**Completed 1 Month Assessment**

**(n=55)**

**Completed Baseline Assessment**

**(n=60)**

**Enrolled**

**(n=60)**
