# Supplementary material for: Burnout among labor and birth providers in northern Tanzania: A mixed-method study
Source: medRxiv. 2023 Jun 3:2023.05.28.23290395. Preprint. [Version 1] doi: 10.1101/2023.05.28.23290395 (PMC10312826; doi:10.1101/2023.05.28.23290395)
Supplement: Supplementary file 2 — S2. Raw data from quantitative provider surveys at four timepoints [file media-2.pdf]

ID	timepoint	dem1	dem2	dem3	dem3_other	dem4	dem5	dem6	dem7	burn1	burn2
1	pre-training	47	1	3	NA	20	15	1	1	1	1
1	mid-training	NA	NA	NA	NA	NA	NA	NA	NA	1	2
1	1 month-training	NA	NA	NA	NA	NA	NA	NA	NA	1	1
1	3 month-training	NA	NA	NA	NA	NA	NA	NA	NA	1	1
2	pre-training	28	1	2	NA	7	5	5	1	1	0
2	mid-training	NA	NA	NA	NA	NA	NA	NA	NA	2	1
2	1 month-training	NA	NA	NA	NA	NA	NA	NA	NA	2	0
2	3 month-training	NA	NA	NA	NA	NA	NA	NA	NA	2	0
3	pre-training	33	1	2	NA	10	4	7	4	0	3
3	mid-training	NA	NA	NA	NA	NA	NA	NA	NA	2	5
3	1 month-training	NA	NA	NA	NA	NA	NA	NA	NA	3	5
3	3 month-training	NA	NA	NA	NA	NA	NA	NA	NA	3	5
4	pre-training	39	1	4	NA	14	6	0.3	1	4	1
4	mid-training	NA	NA	NA	NA	NA	NA	NA	NA	3	0
4	1 month-training	NA	NA	NA	NA	NA	NA	NA	NA	NA	NA
4	3 month-training	NA	NA	NA	NA	NA	NA	NA	NA	0	0
5	pre-training	28	1	2	NA	1	1	1	1	0	1
5	mid-training	NA	NA	NA	NA	NA	NA	NA	NA	3	1
5	1 month-training	NA	NA	NA	NA	NA	NA	NA	NA	NA	NA
5	3 month-training	NA	NA	NA	NA	NA	NA	NA	NA	0	1
6	pre-training	28	1	2	NA	7	1	2	1	4	1
6	mid-training	NA	NA	NA	NA	NA	NA	NA	NA	4	1
6	1 month-training	NA	NA	NA	NA	NA	NA	NA	NA	4	1
6	3 month-training	NA	NA	NA	NA	NA	NA	NA	NA	0	1
7	pre-training	29	1	2	NA	6	1	NA	1	4	1
7	mid-training	NA	NA	NA	NA	NA	NA	NA	NA	4	1
7	1 month-training	NA	NA	NA	NA	NA	NA	NA	NA	0	1
7	3 month-training	NA	NA	NA	NA	NA	NA	NA	NA	0	0
8	pre-training	28	2	2	NA	4	3	3	2	0	0
8	mid-training	NA	NA	NA	NA	NA	NA	NA	NA	2	3
8	1 month-training	NA	NA	NA	NA	NA	NA	NA	NA	0	1
8	3 month-training	NA	NA	NA	NA	NA	NA	NA	NA	4	1
9	pre-training	49	1	2	NA	29	20	6	1	1	2
9	mid-training	NA	NA	NA	NA	NA	NA	NA	NA	1	1
9	1 month-training	NA	NA	NA	NA	NA	NA	NA	NA	1	0
9	3 month-training	NA	NA	NA	NA	NA	NA	NA	NA	0	1
10	pre-training	33	1	4	NA	2	2	2	2	1	2
10	mid-training	NA	NA	NA	NA	NA	NA	NA	NA	1	3
10	1 month-training	NA	NA	NA	NA	NA	NA	NA	NA	0	0
10	3 month-training	NA	NA	NA	NA	NA	NA	NA	NA	0	0
11	pre-training	52	2	4	NA	20	20	3	1	0	0
11	mid-training	NA	NA	NA	NA	NA	NA	NA	NA	0	0
11	1 month-training	NA	NA	NA	NA	NA	NA	NA	NA	1	1
11	3 month-training	NA	NA	NA	NA	NA	NA	NA	NA	1	0
12	pre-training	32	2	2	NA	8	5	7	1	0	0
12	mid-training	NA	NA	NA	NA	NA	NA	NA	NA	1	0
12	1 month-training	NA	NA	NA	NA	NA	NA	NA	NA	0	0
12	3 month-training	NA	NA	NA	NA	NA	NA	NA	NA	0	0
13	pre-training	30	2	2	NA	3	2	1	1	0	0
13	mid-training	NA	NA	NA	NA	NA	NA	NA	NA	3	2
13	1 month-training	NA	NA	NA	NA	NA	NA	NA	NA	1	2
13	3 month-training	NA	NA	NA	NA	NA	NA	NA	NA	0	2
14	pre-training	27	2	2	NA	3	0.3	0.3	1	0	0
14	mid-training	NA	NA	NA	NA	NA	NA	NA	NA	0	0
14	1 month-training	NA	NA	NA	NA	NA	NA	NA	NA	0	0
14	3 month-training	NA	NA	NA	NA	NA	NA	NA	NA	0	0
15	pre-training	39	1	4	NA	5	5	5	1	1	1
15	mid-training	NA	NA	NA	NA	NA	NA	NA	NA	2	1
15	1 month-training	NA	NA	NA	NA	NA	NA	NA	NA	0	1
15	3 month-training	NA	NA	NA	NA	NA	NA	NA	NA	0	1
16	pre-training	53	2	4	NA	25	12	12	1	2	5
16	mid-training	NA	NA	NA	NA	NA	NA	NA	NA	2	3
16	1 month-training	NA	NA	NA	NA	NA	NA	NA	NA	0	3
16	3 month-training	NA	NA	NA	NA	NA	NA	NA	NA	0	1
17	pre-training	34	1	2	NA	12	4	12	1	2	1
17	mid-training	NA	NA	NA	NA	NA	NA	NA	NA	2	1
17	1 month-training	NA	NA	NA	NA	NA	NA	NA	NA	2	3

17	3 month-training	NA	NA	NA	NA	NA	NA	NA	NA	1	0
18	pre-training	28	1	2	NA	6	1	1	1	2	3
18	mid-training	NA	NA	NA	NA	NA	NA	NA	NA	2	3
18	1 month-training	NA	NA	NA	NA	NA	NA	NA	NA	1	3
18	3 month-training	NA	NA	NA	NA	NA	NA	NA	NA	1	5
19	pre-training	34	2	2	NA	11	6	10	2	0	1
19	mid-training	NA	NA	NA	NA	NA	NA	NA	NA	2	3
19	1 month-training	NA	NA	NA	NA	NA	NA	NA	NA	1	2
19	3 month-training	NA	NA	NA	NA	NA	NA	NA	NA	1	1
20	pre-training	52	2	4	NA	28	10	4	1	1	1
20	mid-training	NA	NA	NA	NA	NA	NA	NA	NA	1	1
20	1 month-training	NA	NA	NA	NA	NA	NA	NA	NA	0	1
20	3 month-training	NA	NA	NA	NA	NA	NA	NA	NA	0	1
21	pre-training	32	1	2	NA	9	7	9	1	0	0
21	mid-training	NA	NA	NA	NA	NA	NA	NA	NA	0	1
21	1 month-training	NA	NA	NA	NA	NA	NA	NA	NA	0	1
21	3 month-training	NA	NA	NA	NA	NA	NA	NA	NA	0	0
22	pre-training	25	2	2	NA	3	2	2	2	0	1
22	mid-training	NA	NA	NA	NA	NA	NA	NA	NA	1	2
22	1 month-training	NA	NA	NA	NA	NA	NA	NA	NA	0	1
22	3 month-training	NA	NA	NA	NA	NA	NA	NA	NA	1	1
23	pre-training	26	2	3	NA	1	1	1	1	1	1
23	mid-training	NA	NA	NA	NA	NA	NA	NA	NA	2	1
23	1 month-training	NA	NA	NA	NA	NA	NA	NA	NA	2	1
23	3 month-training	NA	NA	NA	NA	NA	NA	NA	NA	2	1
24	pre-training	27	2	4	NA	2	2	1	1	1	0
24	mid-training	NA	NA	NA	NA	NA	NA	NA	NA	2	1
24	1 month-training	NA	NA	NA	NA	NA	NA	NA	NA	1	1
24	3 month-training	NA	NA	NA	NA	NA	NA	NA	NA	1	1
25	pre-training	NA	2	2	NA	2	2	1	1	0	0
25	mid-training	NA	NA	NA	NA	NA	NA	NA	NA	2	1
25	1 month-training	NA	NA	NA	NA	NA	NA	NA	NA	NA	NA
25	3 month-training	NA	NA	NA	NA	NA	NA	NA	NA	4	1
26	pre-training	32	1	2	NA	10	10	9	1	0	0
26	mid-training	NA	NA	NA	NA	NA	NA	NA	NA	3	1
26	1 month-training	NA	NA	NA	NA	NA	NA	NA	NA	3	1
26	3 month-training	NA	NA	NA	NA	NA	NA	NA	NA	4	1
27	pre-training	30	2	2	NA	10	3	9	1	0	0
27	mid-training	NA	NA	NA	NA	NA	NA	NA	NA	2	1
27	1 month-training	NA	NA	NA	NA	NA	NA	NA	NA	2	0
27	3 month-training	NA	NA	NA	NA	NA	NA	NA	NA	1	1
28	pre-training	52	2	4	NA	17	12	12	1	0	0
28	mid-training	NA	NA	NA	NA	NA	NA	NA	NA	0	0
28	1 month-training	NA	NA	NA	NA	NA	NA	NA	NA	0	0
28	3 month-training	NA	NA	NA	NA	NA	NA	NA	NA	0	0
29	pre-training	29	2	4	NA	2	1	1	1	3	2
29	mid-training	NA	NA	NA	NA	NA	NA	NA	NA	3	3
29	1 month-training	NA	NA	NA	NA	NA	NA	NA	NA	3	3
29	3 month-training	NA	NA	NA	NA	NA	NA	NA	NA	4	3
30	pre-training	45	1	2	NA	18	10	10	1	0	3
30	mid-training	NA	NA	NA	NA	NA	NA	NA	NA	1	0
30	1 month-training	NA	NA	NA	NA	NA	NA	NA	NA	0	0
30	3 month-training	NA	NA	NA	NA	NA	NA	NA	NA	0	0
31	pre-training	32	2	3	NA	8	8	8	1	0	0
31	mid-training	NA	NA	NA	NA	NA	NA	NA	NA	3	3
31	1 month-training	NA	NA	NA	NA	NA	NA	NA	NA	1	1
31	3 month-training	NA	NA	NA	NA	NA	NA	NA	NA	1	1
32	pre-training	37	2	2	NA	8	8	8	2	1	2
32	mid-training	NA	NA	NA	NA	NA	NA	NA	NA	4	3
32	1 month-training	NA	NA	NA	NA	NA	NA	NA	NA	1	1
32	3 month-training	NA	NA	NA	NA	NA	NA	NA	NA	1	1
33	pre-training	28	2	2	NA	6	2	1	3	0	0
33	mid-training	NA	NA	NA	NA	NA	NA	NA	NA	2	0
33	1 month-training	NA	NA	NA	NA	NA	NA	NA	NA	1	1
33	3 month-training	NA	NA	NA	NA	NA	NA	NA	NA	3	0
34	pre-training	34	1	2	NA	11	11	11	1	0	0
34	mid-training	NA	NA	NA	NA	NA	NA	NA	NA	2	5
34	1 month-training	NA	NA	NA	NA	NA	NA	NA	NA	2	0

34	3 month-training	NA	NA	NA	NA	NA	NA	NA	NA	2	0
35	pre-training	29	2	4	NA	3	1	1	2	0	1
35	mid-training	NA	NA	NA	NA	NA	NA	NA	NA	3	5
35	1 month-training	NA	NA	NA	NA	NA	NA	NA	NA	1	3
35	3 month-training	NA	NA	NA	NA	NA	NA	NA	NA	1	1
36	pre-training	43	2	4	NA	20	10	1	4	0	1
36	mid-training	NA	NA	NA	NA	NA	NA	NA	NA	1	1
36	1 month-training	NA	NA	NA	NA	NA	NA	NA	NA	2	3
36	3 month-training	NA	NA	NA	NA	NA	NA	NA	NA	2	3
37	pre-training	26	1	2	NA	6	4	1	2	0	2
37	mid-training	NA	NA	NA	NA	NA	NA	NA	NA	3	3
37	1 month-training	NA	NA	NA	NA	NA	NA	NA	NA	NA	NA
37	3 month-training	NA	NA	NA	NA	NA	NA	NA	NA	1	4
38	pre-training	30	2	3	NA	6	4	4	2	1	2
38	mid-training	NA	NA	NA	NA	NA	NA	NA	NA	3	4
38	1 month-training	NA	NA	NA	NA	NA	NA	NA	NA	2	1
38	3 month-training	NA	NA	NA	NA	NA	NA	NA	NA	0	1
39	pre-training	34	2	2	NA	8	7	7	1	4	5
39	mid-training	NA	NA	NA	NA	NA	NA	NA	NA	2	5
39	1 month-training	NA	NA	NA	NA	NA	NA	NA	NA	1	3
39	3 month-training	NA	NA	NA	NA	NA	NA	NA	NA	1	2
40	pre-training	30	1	2	NA	10	9	9	2	2	6
40	mid-training	NA	NA	NA	NA	NA	NA	NA	NA	2	5
40	1 month-training	NA	NA	NA	NA	NA	NA	NA	NA	2	1
40	3 month-training	NA	NA	NA	NA	NA	NA	NA	NA	3	3
41	pre-training	35	2	4	NA	2	2	1	1	1	3
41	mid-training	NA	NA	NA	NA	NA	NA	NA	NA	3	5
41	1 month-training	NA	NA	NA	NA	NA	NA	NA	NA	1	3
41	3 month-training	NA	NA	NA	NA	NA	NA	NA	NA	1	2
42	pre-training	32	2	2	NA	5	4	5	1	1	1
42	mid-training	NA	NA	NA	NA	NA	NA	NA	NA	3	2
42	1 month-training	NA	NA	NA	NA	NA	NA	NA	NA	1	0
42	3 month-training	NA	NA	NA	NA	NA	NA	NA	NA	1	0
43	pre-training	29	1	2	NA	8	6	2	1	1	1
43	mid-training	NA	NA	NA	NA	NA	NA	NA	NA	1	1
43	1 month-training	NA	NA	NA	NA	NA	NA	NA	NA	1	1
43	3 month-training	NA	NA	NA	NA	NA	NA	NA	NA	0	1
44	pre-training	32	2	2	NA	6	3	1	1	1	1
44	mid-training	NA	NA	NA	NA	NA	NA	NA	NA	2	1
44	1 month-training	NA	NA	NA	NA	NA	NA	NA	NA	0	0
44	3 month-training	NA	NA	NA	NA	NA	NA	NA	NA	2	1
45	pre-training	37	1	2	NA	13	7	8	1	0	0
45	mid-training	NA	NA	NA	NA	NA	NA	NA	NA	1	1
45	1 month-training	NA	NA	NA	NA	NA	NA	NA	NA	0	0
45	3 month-training	NA	NA	NA	NA	NA	NA	NA	NA	0	2
46	pre-training	25	2	2	NA	4	2	1	2	0	2
46	mid-training	NA	NA	NA	NA	NA	NA	NA	NA	4	4
46	1 month-training	NA	NA	NA	NA	NA	NA	NA	NA	2	2
46	3 month-training	NA	NA	NA	NA	NA	NA	NA	NA	2	2
47	pre-training	46	2	2	NA	11	11	11	1	4	3
47	mid-training	NA	NA	NA	NA	NA	NA	NA	NA	1	1
47	1 month-training	NA	NA	NA	NA	NA	NA	NA	NA	0	1
47	3 month-training	NA	NA	NA	NA	NA	NA	NA	NA	0	0
48	pre-training	42	2	4	NA	14	7	14	1	0	0
48	mid-training	NA	NA	NA	NA	NA	NA	NA	NA	0	0
48	1 month-training	NA	NA	NA	NA	NA	NA	NA	NA	0	0
48	3 month-training	NA	NA	NA	NA	NA	NA	NA	NA	0	0
49	pre-training	31	2	4	NA	2	2	2	1	0	0
49	mid-training	NA	NA	NA	NA	NA	NA	NA	NA	0	0
49	1 month-training	NA	NA	NA	NA	NA	NA	NA	NA	NA	NA
49	3 month-training	NA	NA	NA	NA	NA	NA	NA	NA	NA	NA
50	pre-training	29	1	1	NA	9	5	9	2	0	0
50	mid-training	NA	NA	NA	NA	NA	NA	NA	NA	0	0
50	1 month-training	NA	NA	NA	NA	NA	NA	NA	NA	0	0
50	3 month-training	NA	NA	NA	NA	NA	NA	NA	NA	0	0
51	pre-training	35	1	2	NA	8	6	8	1	1	1
51	mid-training	NA	NA	NA	NA	NA	NA	NA	NA	3	1
51	1 month-training	NA	NA	NA	NA	NA	NA	NA	NA	1	1

51	3 month-training	NA	NA	NA	NA	NA	NA	NA	NA	1	1
52	pre-training	36	1	2	NA	11	10	11	1	1	1
52	mid-training	NA	NA	NA	NA	NA	NA	NA	NA	1	1
52	1 month-training	NA	NA	NA	NA	NA	NA	NA	NA	0	1
52	3 month-training	NA	NA	NA	NA	NA	NA	NA	NA	NA	NA
53	pre-training	NA	1	2	NA	30	10	30	1	0	0
53	mid-training	NA	NA	NA	NA	NA	NA	NA	NA	2	1
53	1 month-training	NA	NA	NA	NA	NA	NA	NA	NA	0	3
53	3 month-training	NA	NA	NA	NA	NA	NA	NA	NA	0	1
54	pre-training	40	1	2	NA	8	4	5	1	4	0
54	mid-training	NA	NA	NA	NA	NA	NA	NA	NA	3	1
54	1 month-training	NA	NA	NA	NA	NA	NA	NA	NA	3	1
54	3 month-training	NA	NA	NA	NA	NA	NA	NA	NA	3	1
55	pre-training	29	2	4	NA	3	0.25	0.25	1	0	0
55	mid-training	NA	NA	NA	NA	NA	NA	NA	NA	1	0
55	1 month-training	NA	NA	NA	NA	NA	NA	NA	NA	2	0
55	3 month-training	NA	NA	NA	NA	NA	NA	NA	NA	2	0
56	pre-training	57	2	4	NA	10	6	6	1	0	2
56	mid-training	NA	NA	NA	NA	NA	NA	NA	NA	2	2
56	1 month-training	NA	NA	NA	NA	NA	NA	NA	NA	0	1
56	3 month-training	NA	NA	NA	NA	NA	NA	NA	NA	2	1
57	pre-training	40	1	2	NA	15	7	15	1	0	0
57	mid-training	NA	NA	NA	NA	NA	NA	NA	NA	1	5
57	1 month-training	NA	NA	NA	NA	NA	NA	NA	NA	2	3
57	3 month-training	NA	NA	NA	NA	NA	NA	NA	NA	4	1
58	pre-training	55	1	2	NA	25	8	25	1	0	0
58	mid-training	NA	NA	NA	NA	NA	NA	NA	NA	0	0
58	1 month-training	NA	NA	NA	NA	NA	NA	NA	NA	0	0
58	3 month-training	NA	NA	NA	NA	NA	NA	NA	NA	0	0
59	pre-training	28	1	2	NA	9	4	4	1	0	0
59	mid-training	NA	NA	NA	NA	NA	NA	NA	NA	0	0
59	1 month-training	NA	NA	NA	NA	NA	NA	NA	NA	0	0
59	3 month-training	NA	NA	NA	NA	NA	NA	NA	NA	0	0
60	pre-training	38	1	2	NA	8	4	2	1	0	0
60	mid-training	NA	NA	NA	NA	NA	NA	NA	NA	1	2
60	1 month-training	NA	NA	NA	NA	NA	NA	NA	NA	0	0
60	3 month-training	NA	NA	NA	NA	NA	NA	NA	NA	0	0

dem1 How old are you?
dem2 What is your gender [1=Female, 2=Male]
dem3 What is your clinical training [1=Midwife, 2=Nurse midwife, 3=Clinical officer, 4=Medical doctor, 5=Other]
dem4 How many years has it been since you completed your clinical schooling?
dem5 How many years have you been providing care in labor and delivery?
dem6 How many years have you been working at the clinic/hospital where youre currently employed?
dem7 In the last year, how many trainings have you completed that included content about labor and delivery for women with HIV
burn1 Using your own definition of burnout, please select one of the following responses [0= I enjoy my work. I have no symptoms of burnout. 1= Occasionally I am under stress, and I don't always have as much energy as I once did, but I don't feel burned out. 2= I am definitely burning out and have one or more symptoms of burnout, such as physical and emotional exhaustion. 3= The symptoms of burnout I am experiencing won't go away. I think about frustrations at work a lot. 5= I feel completely burned out and often wonder if I can go on. I am at the point where I may need some changes or may need to seek some sort of help.]
burn2 How often do you feel youve become more callous toward people since you took this job because of burnout? [0= Never, 1= A few times a year or less, 2= Once a month or less, 3= A few times a month, 4= Once a week, 5= A few times a week, 6= Every day]
